# Neuregulin attenuates diaphragm muscle protein degradation via an intricate signaling network

**DOI:** 10.3389/fphys.2026.1846390

**Published:** 2026-07-14

**Authors:** Nathan J. Hellyer, Heather M. Gransee, Gary C. Sieck, Carlos B. Mantilla

**Affiliations:** 1 Department of Physical Medicine & Rehabilitation, Mayo Clinic, Rochester, MN, United States; 2 Department of Anesthesiology & Perioperative Medicine, Mayo Clinic, Rochester, MN, United States; 3 Department of Physiology & Biomedical Engineering, Mayo Clinic, Rochester, MN, United States

**Keywords:** epidermal growth factor, heregulin, muscle atrophy, protein balance, skeletal muscle, trophic factor

## Abstract

**Introduction:**

The nerve-derived growth factor neuregulin (NRG) plays a role in the regulation of skeletal muscle mass through Akt and mTOR signaling transduction pathways that regulate both protein synthesis and degradation. We previously reported that NRG increases muscle protein synthesis (~20%) in a PI3 kinase (PI3K)/Akt-dependent manner. However, the effects of NRG on protein degradation are still poorly understood and are needed to elucidate the role of NRG in the maintenance of skeletal muscle protein balance.

**Methods:**

Neonatal diaphragm muscle *ex vivo* preparations were pharmacologically treated with NRG and pharmacological inhibitors of PI3K (LY294002, 50 μM), MEK (PD98059, 50 μM) or mTOR (rapamycin, 100 nM). Tyrosine release from muscle was used as a surrogate measure of protein degradation.

**Results:**

We report that basal protein degradation in the neonatal rat diaphragm muscle is significantly reduced by NRG treatment (19%). Basal protein degradation was increased following treatment with inhibitors of PI3K, MEK or mTOR, with inhibition of each pathway sufficient to increase basal protein degradation greater than 30%. Importantly, NRG treatment in the presence of each of these inhibitors blunts the increase in protein degradation induced by inhibition of PI3K, MEK or mTOR. NRG effects were significantly blunted by rapamycin (p < 0.05 compared to NRG alone), but not by LY294002 or PD98059.

**Discussion:**

We suggest that both the PI3K/Akt and MAP kinase pathways are important for NRG effects on protein degradation, but that mTOR may be a critical modulator of these effects and thus of protein balance in skeletal muscle.

## Introduction

The nerve-derived growth factor neuregulin (NRG) regulates nerve-muscle communication, particularly during growth and development (Fischbach and Rosen, 1997; Loeb et al., 1999). Disrupting NRG signaling in genetically modified mice causes severe impairments in cardiac muscle development and is fatal (Meyer and Birchmeier, 1995). In skeletal muscle, NRG stimulates myogenin expression in myoblasts which may induce myotube differentiation (Kim et al., 1999; Ford et al., 2003). In addition, NRG appears to provide neurotrophic support important for the maintenance of skeletal muscle mass by increasing protein synthesis via a PI3K/Akt-dependent pathway (Hellyer et al., 2006). The present study evaluates the role of NRG on skeletal muscle protein degradation. It is well established that PI3K/Akt, MAPK and mTOR are core mediators of NRG signaling (Zeng et al., 2024; Trombetta et al., 2025). NRG is a particularly strong activator of the PI3K pathway, as the NRG ErbB3 co-receptor has six docking sites of the p85 subunit of PI3K (Hellyer et al., 1998). This PI3K/Akt pathway stimulates protein translation via downstream activation of mTOR and S6 ribosomal proteins (Magnuson et al., 2012; Yoshida and Delafontaine, 2020). NRG may also activate MAPK signaling cascades that regulate cell growth and proliferation (Vijapurkar et al., 1998; Ravichandran, 2001). The exact contribution of the PI3K and MAPK pathways to the NRG effects on the regulation of protein degradation is not clear. However, the role of NRG on protein degradation, and thus in the maintenance of protein balance is still poorly understood, yet this information is essential to understand NRG effects on skeletal muscle mass.

The maintenance of skeletal muscle mass depends not only on anabolic processes but also on the modulation of protein degradation rates. In various tissues, including skeletal muscle, the PI3K/Akt pathway not only promotes protein synthesis but may also inhibit protein degradation through FOXO suppression and inhibition of ubiquitin ligase synthesis (Yoshida and Delafontaine, 2020). Therefore, it is possible that NRG may both promote protein synthesis and inhibit protein degradation. The importance of NRG signaling in regulating protein degradation is supported by the observation that denervation, which removes nerve-derived growth factors including NRG, leads to muscle atrophy primarily due to increased protein degradation (Argadine et al., 2011; Zhang et al., 2025). Corroborating this is the observation that NRG reduces sepsis induced autophagy in cultured L6 muscle cells and rat diaphragm in an Akt dependent manner (Yin et al., 2022). Our hypothesis is that NRG reduces diaphragm muscle protein degradation. The present study directly evaluates the effects of NRG on skeletal muscle protein degradation (measured by tyrosine release in the presence of protein synthesis inhibitors) and explores the contribution of the PI3K/Akt, MAPK and mTOR to NRG effects.

## Materials and methods

### Diaphragm muscle samples

The “Guide for the Care and Use of Laboratory Animals: Eighth Edition (National Research Council (U.S.). Committee for the Update of the Guide for the Care and Use of Laboratory Animals. et al., 2011)” was followed for all methodological procedures and the Institutional Animal Care and Use Committee at the Mayo Clinic reviewed and approved the use of animals for the proposed experiments (Protocol A19909). Sprague Dawley rat pups, sourced from timed-pregnant females (gestational day 15; Charles River Laboratories, Wilmington, DE), were used for experiments. Three-day-old unanesthetized rat pups were euthanized by immediate decapitation in accordance with published guidelines (National Research Council (U.S.). Committee for the Update of the Guide for the Care and Use of Laboratory Animals. et al., 2011). Neonatal diaphragm muscles were utilized for protein degradation experiments due to their extremely thin muscle structure allowing for the ease of NRG and inhibitor permeability (Hellyer et al., 2006). The costal diaphragm was immediately removed from sacrificed animals (an average of 13 pups from 12 litters; n = 150), rinsed in Krebs-Ringer buffer [NaCl (118.3 mM), KCl (4.7 mM), CaCl_2_ (2.5 mM), KH_2_PO_4_ (1.2 mM), MgSO_4_ (1.2 mM), NaHCO_3_ (25 mM), dextrose (13.9 mM), pH = 7.4] and promptly pinned at approximate resting length in a twelve well culture plate chamber whose bottom was lined with silicone rubber (Sylgard; Dow Corning, Midland, MI). The diaphragm muscle was incubated in 1.0 ml Krebs-Ringer buffer supplemented with 0.5 mM cycloheximide at 37 °C with 5% CO_2_ for 30 minutes. Subsequently samples were treated with either phosphate-buffered saline (PBS) or pharmacological inhibitor dissolved in PBS for 30 minutes. Inhibitors were originally dissolved in ethanol to 5mM concentrations before being diluted in PBS to the final concentrations indicated:PD98059 (50 μM) (Alessi et al., 1995), LY 294002 (50 μM) (Blommaart et al., 1997), and rapamycin (100 nM) (Brunn et al., 1996; Hay and Sonenberg, 2004) (Cell Signaling Technology, Danvers, MA). PD98059 was used to inhibit MEK, LY294002 was used to inhibit PI3K, and rapamycin was used to inhibit mTOR. Following 30 minutes of inhibitor treatment, recombinant NRG (40 nM, Heregulin β177–244, Upstate Biotechnology, Waltham, MA) was added to each well for 30 minutes (1 ml total volume) [6]. This buffer was then removed, and the muscle preps were allowed to incubate for a further three hours in 1 ml of fresh Krebs-Ringer buffer containing the appropriate inhibitor and/or NRG treatment. After three hours of incubation in buffer/inhibitor/NRG solution, experiments were terminated by quickly removing all of the buffer solution which was then analyzed for tyrosine content. The diaphragm muscle was rinsed with PBS, blotted dry, and promptly weighed to determine muscle mass.

### Analysis of protein degradation

Tyrosine release from muscle was used as a surrogate measure of protein degradation (Argadine et al., 2009; Waalkes and Udenfriend, 1957). Buffer from muscles preparations was added to an equal volume of nitrosonaphthol-nitric acid reagent and incubated at 55 °C for 30 min. The nitrosonapthol-nitric acid reagent was made by premixing equal volumes of a nitric acid solution (98:2, 20% nitric acid and 2.5% sodium nitrite) to 0.5% nitrosonapthol (GFS Chemicals. Powell, OH). The fluorescent tyrosine derivative generated from the reaction was extracted by adding five times the volume of ethylene dichloride (Sigma, St. Louis, MO). Three replicates were performed for each reaction with samples pipetted into 96 well microtiter plates. Simultaneous reactions employing known L-tyrosine (Sigma, St. Louis, MO) concentrations were used as reference standards. Spectrophotofluorimetry (Molecular Devices, San Jose, CA) was used to quantitate tyrosine concentration (excitation at 460 nm and emission at 570–630 nm). Tyrosine release was expressed relative to diaphragm muscle mass (nmol/μg).

### Statistical analysis

Data were analyzed using JMP 18.1.1 (SAS Institute Inc., Cary, NY, USA). Tyrosine release was analyzed using a mixed linear model with NRG treatment and pharmacological inhibitor treatment as fixed effects and rat litter as random effect. When indicated, *post-hoc* analysis of least squares means differences was conducted using the Tukey-Kramer Honestly Significant Difference test to determine significant differences between specific treatment groups. A p-value less than 0.05 was considered statistically significant. All experimental data are reported as mean ± standard deviation.

## Results

**Figure 1:**
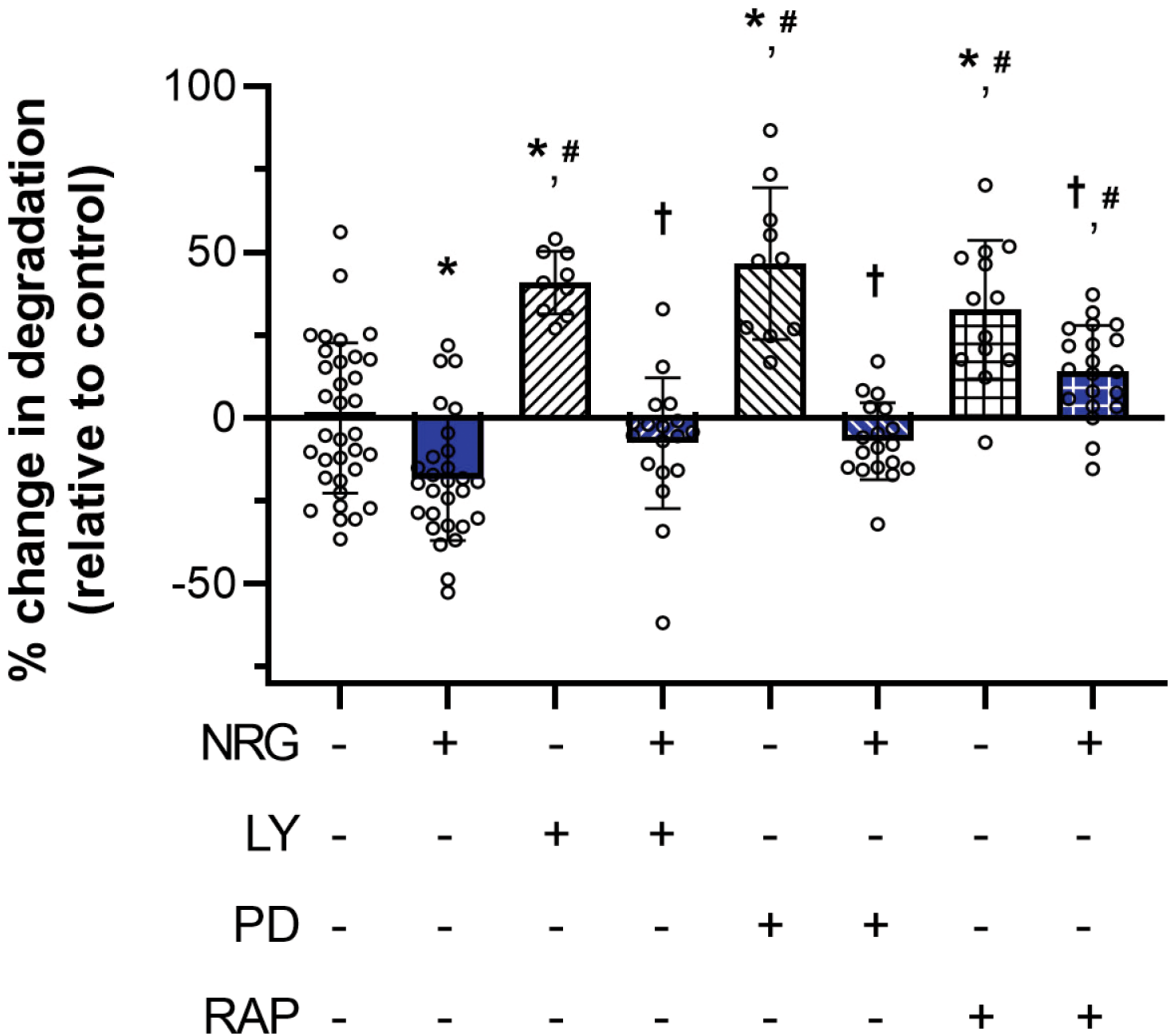
Relative change in tyrosine release from neonatal rat diaphragm muscle preparations following neuregulin or inhibitor treatment compared to control (basal protein degradation). Each open circle represents a single preparation. *p < 0.05 vs. control; ^#^p < 0.05 vs. NRG treatment, ^†^vs. corresponding inhibitor without NRG co-treatment.

Tyrosine release from muscle was used as a surrogate measure of protein degradation. Fresh *ex vivo* diaphragm muscle preparations were analyzed for protein degradation following NRG treatment with various pharmacological inhibitors in the presence of cycloheximide (**Table 1**). Since tyrosine is not synthesized or degraded in skeletal muscle, its release reflects protein degradation when protein synthesis is inhibited via cycloheximide (Argadine et al., 2011). The percentage change in degradation, as measured by the change in tyrosine release compared to control treatment, was calculated for each treatment group (**Figure 1**).

**Table 1:** Tyrosine release from skeletal muscle (nmol/mg muscle ± S.D).

Inhibitor	Vehicle		Neuregulin	
No inhibitor	n = 34	0.58 ± 0.13	n = 28	0.47 ± 0.11 *
LY294002	n = 9	0.80 ± 0.06 *,#	n = 18	0.53 ± 0.11 †
PD98059	n = 10	0.82 ± 0.15 *,#	n = 18	0.54 ± 0.07 †
Rapamycin	n = 13	0.76 ± 0.14 *,#	n = 20	0.66 ± 0.08 †,#

The number of individual preparations for each condition is specified and reflect pups obtained from 12 litters (on average 13 pups/litter).

*p < 0.05 vs. control (vehicle, no inhibitor group); ^#^p < 0.05 vs. NRG treatment, ^†^vs. corresponding inhibitor without NRG co-treatment.

There was a significant effect of neuregulin treatment (F_1,12_ = 39, p < 0.01), inhibitor treatment (F_3,69_ = 11, p < 0.01), and their interaction (F_3,64_ = 5, p < 0.01) on tyrosine release per muscle weight (**Table 1**). There was no evidence of a litter effect in the mixed linear model (Wald p-value = 0.09). *Post-hoc* analysis showed that NRG treatment significantly reduced protein degradation by 19% compared to control treatment (**Figure 1**; p < 0.05).

All three pharmacological inhibitors resulted in an increase in protein degradation relative to the control condition (**Figure 1**). When the PI 3-kinase enzyme was inhibited by the LY294002 compound there was a 41% increase in protein degradation (p < 0.05 compared to control). Likewise, protein degradation increased when diaphragm muscle was treated with the MEK inhibitor PD98059 or the mTOR inhibitor rapamycin (**Figure 1**), with protein degradation increasing by 46% and 33% relative to control, respectively (p < 0.05 in both cases).

Following inhibitor treatment, NRG effects to reduce protein degradation were blunted or reversed (**Figure 1**). LY294002 increased protein degradation by 41% relative to control. In the presence of LY294002, NRG reduced protein degradation by only 8%, significantly attenuating the NRG-mediated effect following PI3-kinase inhibition (p < 0.05). Similarly, PD98059 increased protein degradation by 46% relative to control. In the presence of PD98059, NRG reduced protein degradation by only 7%, significantly attenuating the NRG effect following MEK inhibition (p < 0.05).

Thus, following treatment with either of these inhibitors, NRG restored protein degradation to basal conditions (p > 0.05 vs. control in both cases), and there was no significant difference compared to NRG alone (p > 0.05 vs. control in both cases). NRG treatment in the presence of rapamycin reduced protein degradation from 33% relative to control with rapamycin alone to 14% (p < 0.05), significantly blunting the effect of mTOR inhibition on protein degradation. Importantly, the effect of NRG to reduce protein degradation was significantly blunted by rapamycin treatment, as seen by NRG treatment in the presence of rapamycin causing significantly greater protein degradation than NRG treatment alone (p < 0.05).

## Discussion

**Figure 2:**
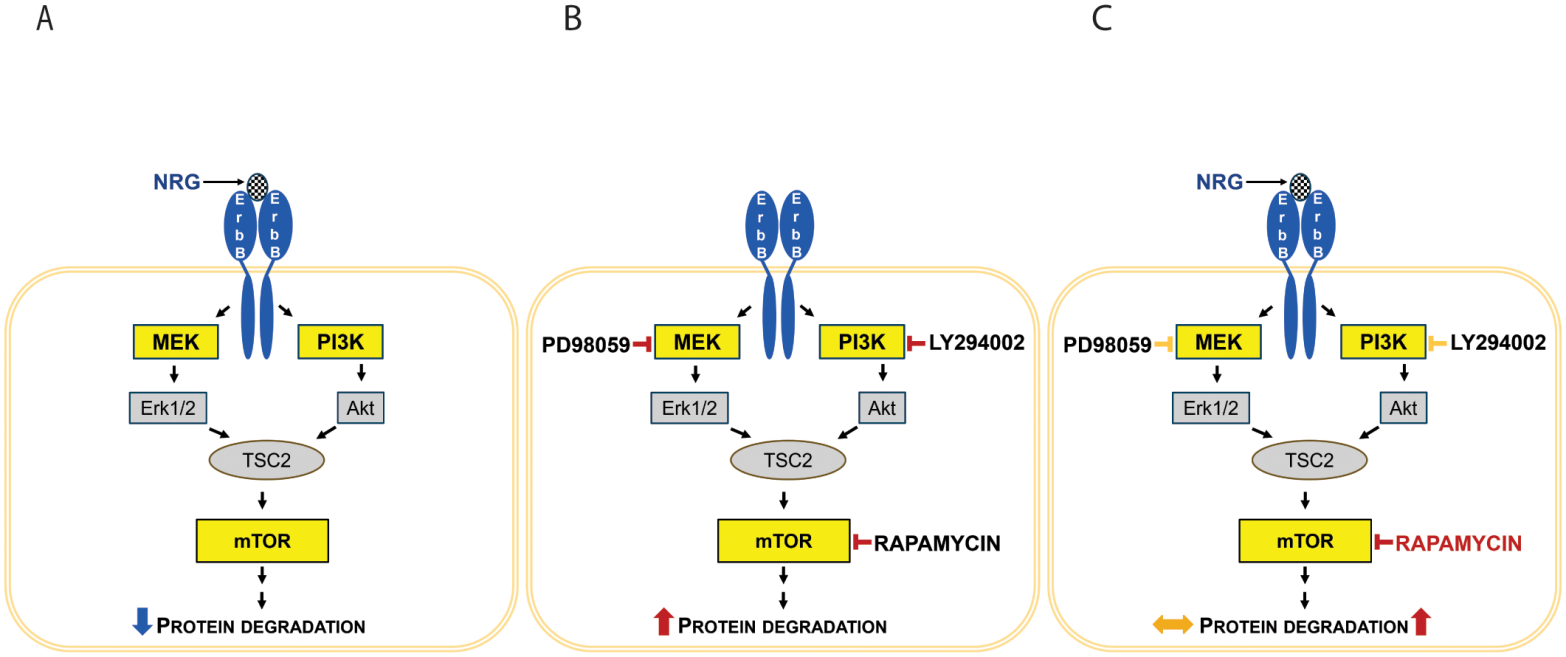
Neuregulin signaling pathways and their effects on protein degradation. **(A)** Neuregulin (NRG) reportedly signals through dimerized ErbB family receptors to activate previously characterized downstream pathways, including MEK and PI3K. NRG treatment reduced protein degradation. **(B)** Pharmacological inhibition of MEK (PD98059), PI3K (LY294002), or mTOR (rapamycin) leads to increased protein degradation. **(C)** The reduction in protein degradation with NRG treatment is unchanged in the presence of either pharmacological inhibition of MEK (PD98059) or PI3K (LY294002). Pharmacological inhibition of mTOR (rapamycin) blunts the NRG induced decrease in protein degradation.

This study investigated the effects of NRG on skeletal muscle protein degradation using *ex vivo* diaphragm preparations and highlights the potential of NRG as a modulator of muscle protein homeostasis, with potential therapeutic application. NRG was previously shown to support the maintenance of skeletal muscle mass by increasing protein synthesis via a PI3K/Akt-dependent pathway (Hellyer et al., 2006). The present study shows that NRG treatment decreases protein degradation, an effect that was blunted by mTOR inhibition but not by inhibition of PI3K or MEK alone (**Figure 2**). Of note, the increased degradation induced by inhibition of PI3K, MEK or mTOR was blunted significantly by NRG treatment. Together, these results support an important role for NRG in maintenance of protein balance and presumably skeletal muscle mass.

NRG binds to various co-receptor combinations of the ErbB family (Britsch, 2007). The ErbB2/ErbB3 heterodimer induces myogenin expression in myotubes (Altiok et al., 1995; Moscoso et al., 1995). ErbB3 is significantly important because, among the four ErbB receptor tyrosine kinase family members, it possesses six PI3K binding sites. Phosphorylated site pairs cooperatively bind to the p85 subunit of PI3K (Hellyer et al., 1998, 2001). This PI3K/Akt pathway stimulates protein translation via downstream activation of mTOR and S6 ribosomal proteins (Magnuson et al., 2012). ErbB3 also contains Shc binding motifs (Vijapurkar et al., 1998). Shc is an intracellular second messenger protein that activates the MAPK signaling cascade and helps regulate cell growth and proliferation (Ravichandran, 2001). The thin neonatal diaphragm muscle *ex vivo* preparations allow us to investigate whole tissue response in an isolated and controlled manner, offering a physiological advantage over cell culture preparations. Protein degradation, measured as tyrosine release under cycloheximide-inhibited protein synthesis (Argadine et al., 2011; Gomes-Marcondes et al., 2002), was significantly reduced (18% compared to control) by NRG treatment. Reduced protein degradation when taken in consideration of previous work where NRG was observed to also increase protein synthesis (Hellyer et al., 2006), suggests NRG promotes a net protein balance. In contrast, pharmacological inhibition of PI3K (LY294002), MEK (PD98059), or mTOR (rapamycin) each induced an increase in protein breakdown, increasing protein degradation by 41%, 46%, and 33% relative to control, respectively. In the presence of PI3K and MEK inhibition, NRG reduced protein degradation; however, in the presence of mTOR inhibition, NRG did not reduce protein degradation back to the level of the control group. These findings support a downstream role for the mTOR pathway in mediating NRG’s effects on muscle protein degradation. We acknowledge that using cycloheximide as a protein synthesis inhibitor, despite its global employment in protein degradation experiments, may have off target effects on degradation pathways (Li et al., 2021). Despite this limitation, all conditions we tested were under the same cycloheximide treatment minimizing the overall influence in our interpretations.

The PI3K/Akt pathway is a well-established regulator of protein degradation in skeletal muscle (Argadine et al., 2011; Yoshida and Delafontaine, 2020; Feng et al., 2022). Activation of this pathway typically leads to the phosphorylation and subsequent nuclear exclusion of FOXO transcription factors (Yoshida and Delafontaine, 2020), preventing the transcription of genes encoding ubiquitin ligases, key enzymes involved in protein degradation (Bodine et al., 2001; Sandri et al., 2004). Our findings support this established role: inhibition of PI3K using LY294002 resulted in a significant increase in protein degradation (41% compared to control), consistent with reduced FOXO phosphorylation and increased ubiquitin ligase activity (Yoshida and Delafontaine, 2020). Furthermore, the partial reversal of this effect by NRG treatment (8% compared to control) suggests that NRG’s ability to reduce protein degradation is at least partially mediated through PI3K/Akt activation (Hellyer et al., 2001, 2006). The incomplete reversal by NRG indicates that other pathways also contribute to NRG’s effects on protein degradation, possibly because of Akt mediated interactions with lysosomal pathways (Palmieri et al., 2017).

While the precise role of the MAPK/Erk pathway in skeletal muscle protein degradation remains less well-defined compared to PI3K/Akt, our findings suggest a significant regulatory influence. Treatment with the MEK inhibitor PD98059 resulted in a substantial increase in protein degradation (46% compared to control), indicating that MEK activity is necessary for maintaining basal levels of protein degradation. This observation is consistent with previous findings demonstrating MEK’s importance in insulin-mediated caspase reduction in L6 myotubes (Gao et al., 2008), suggesting a potential link between MAPK signaling, apoptosis, and protein degradation. Furthermore, the attenuation of NRG’s protein degradation-reducing effect by PD98059 (to 7% compared to control) suggests that the MEK pathway contributes to NRG’s overall regulatory effects on muscle protein degradation. In contrast, the parallel p38 MAPK pathway inhibition is associated with the increased degradation of protein aggregates (Leestemaker et al., 2017). Future studies should investigate the specific downstream targets of MAPK involved in the regulation of protein degradation. The observed link between MEK and caspase reduction in skeletal muscle models (Gao et al., 2008) further suggests a potential interplay between MAPK signaling and protein degradation in the diaphragm.

It is well recognized that mTOR is a central regulator of muscle protein homeostasis, playing a critical role in both protein synthesis and autophagy (Roberts et al., 2023; Zhao et al., 2016, 2015). mTORC1, a key mTOR complex, promotes protein synthesis while suppressing autophagy (Han et al., 2022; Roberts et al., 2023; Schiaffino et al., 2021). Inhibition of mTOR with rapamycin significantly increased protein degradation (33% compared to control) in a three hour time which is consistent with prior observations (Zhao et al., 2016). Consistent with this is rapamycin’s lessening of NRG’s effects on basal protein degradation. This finding is consistent with mTOR’s role in regulating muscle autophagy through the phosphorylation and inhibition of Ulk1, a key autophagy-initiating kinase (Kim et al., 2011). NRG inhibition of Ulk1, through mTOR activation, might inhibit the first step of sarcomere disassembly which is required to provide substrate to the proteosome for bulk degradation (Kim et al., 2011). The continued activation of mTOR may further inhibit the process of protein degradation through activation of ubiquitin ligases (Sandri et al., 2004), albeit likely on a more extended time frame than what we analyzed in our study.

The reduction of NRG’s effect by rapamycin suggests that mTOR is a downstream mediator of NRG’s influence on protein degradation. It is plausible that TSC1/TSC2 may be a coordinating sensor for mTOR activity downstream of PI 3 kinase and MEK (Wang et al., 2025). The potential role of the TSC1/TSC2 complex as a coordinating sensor for mTOR activity warrants significant attention (Dibble and Cantley, 2015; Gupta et al., 2022). Given the both PI3K/Akt (via Akt-mediated phosphorylation of TSC2) and MAPK/Erk pathways are known to regulate TSC1/TSC2 (Wang et al., 2025), it is plausible that NRG, by activating both PI3K and MEK, converges on TSC1/TSC2 to modulate mTOR activity, thereby, orchestrating its effects on protein degradation warranting further study.

## Limitations

- The *ex vivo* diaphragm model provides control over experimental conditions including permeability of pharmacological treatments but lacks systemic interactions that may influence muscle protein balance *in vivo* (e.g., other neurohumoral regulators). In addition, tyrosine release is an accepted surrogate measure of global protein degradation but does not allow for the analysis of specific proteins (e.g., myofibrillar proteins) or pathways. Future studies are needed to evaluate *in vivo* effects of NRG treatment on skeletal muscle protein balance and mass.
- Pharmacological inhibitors, such as LY294002, PD98059, and rapamycin, may exhibit off-target effects despite their reported selectivity at the concentrations used. Based on these findings, however, future studies may target these pathways using alternative approaches to elucidate NRG effects on protein balance in skeletal muscle (e.g., genetic manipulation). The present study leveraged neonatal preparations and thus generalizability of these findings to adult and/or aging skeletal muscles needs direct validation.

## Data Availability

The raw data supporting the conclusions of this article will be made available by the authors, without undue reservation.
